# Eye-openers for advanced management of elderly-onset rheumatoid arthritis

**DOI:** 10.1093/rheumatology/keaf432

**Published:** 2025-08-09

**Authors:** Hamit Harun Dag, Selinde V J Snoeck Henkemans, Agnes E M Looijen, Judith W Heutz, Radboud J E M Dolhain, Pascal H P de Jong

**Affiliations:** Department of Rheumatology, Erasmus MC, Rotterdam, The Netherlands; Department of Rheumatology, Erasmus MC, Rotterdam, The Netherlands; Department of Rheumatology, Erasmus MC, Rotterdam, The Netherlands; Department of Rheumatology, Erasmus MC, Rotterdam, The Netherlands; Department of Rheumatology, Erasmus MC, Rotterdam, The Netherlands; Department of Rheumatology, Erasmus MC, Rotterdam, The Netherlands

**Keywords:** rheumatoid arthritis, DMARD, patient-reported outcome measures, elderly-onset rheumatoid arthritis

## Abstract

**Objectives:**

To investigate whether clinical outcomes and patient-reported outcomes (PROs) differ over 2 years based on the age of onset of RA.

**Methods:**

All RA patients from the tREACH trial, a multicentre, stratified, single-blinded trial with a treat-to-target management approach and a fixed medication protocol were included. The age of disease onset was categorized into young-onset RA (YORA) (<45 years, *n* = 119), middle-aged onset RA (MORA) (45–65 years, *n* = 208) and elderly-onset RA (EORA) (>65 years, *n* = 98) at the time of diagnosis. Mixed models were used to compare clinical outcomes and PROs over time. The following PROs were included: pain (Numeric Rating Scale), fatigue (visual analogue scale), functional ability (HAQ-Disability Index, HAQ-DI), quality of life (European Quality of Life 5-Dimensions 3-Levels, EQ-5D-3L), and possible depression (Hospital Anxiety and Depression Scale-Depression, HADS-D) or anxiety disorder (HADS-Anxiety, HADS-A).

**Results:**

At diagnosis, EORA patients had more swollen joints, erosions and comorbidities than younger patients. However, disease activity remained similar across age groups at diagnosis and over time. After 2 years of follow-up, bDMARD usage was 30%, 30% and 15% in YORA, MORA and EORA patients, respectively. EORA patients also experienced less pain and fatigue over time compared with YORA patients [1 (95% CI 0.5–1.6) and 17.3 mm (11.3–23.4) lower] and MORA patients [0.6 (0.1–1) and 5.8 mm (0.7–10.9) lower]. No other clinically relevant PRO differences were observed.

**Conclusion:**

Despite unfavourable prognostic factors at diagnosis, EORA patients have similar outcomes compared with their younger counterparts if a treat-to-target management approach is applied. Notably, fewer EORA patients required bDMARDs to reach the same treatment target.

Rheumatology key messages

- Elderly-onset RA (EORA) patients have similar outcomes compared with younger counterparts under a treat-to-target management approach.
- We encourage striving for a treat-to-target management approach in EORA patients, irrespective of the number of comorbidities.
- Comorbidities should be considered when choosing DMARD treatment in shared decision-making with the patients as well as the overall risk–benefit ratio.

## Introduction

At diagnosis, one-third of patients with RA are older than 65 years, also known as elderly-onset RA (EORA) [1]. Our current knowledge about EORA patients is mainly based on (retrospective) cohort studies. In addition, EORA patients are often underrepresented in clinical trials, while they are the backbone of our management recommendations [2, 3]. In daily practice, rheumatologists tend to treat EORA patients less aggressively due to their advanced age and multiple comorbidities [4–8]. This raises the question whether current management recommendations also apply to EORA patients.

Previous literature has shown that EORA patients have a different clinical presentation compared with young-onset RA (YORA), diagnosis <45 years, and middle-aged onset RA (MORA) patients, diagnosis between 45 and 65 years. At presentation, EORA patients have a longer symptom duration, a more acute onset of symptoms, higher inflammation markers and more often erosive disease [4, 5, 9–12]. With respect to the presence of RF and ACPA and their titres, conflicting results were found [10, 13]. If EORA patients have a different clinical presentation, including the presence of poor prognostic factors, then the management approach might also need to be tailored to this subgroup of RA patients.

Currently, EORA patients are more often treated with glucocorticoids (GC) and less often with a combination of DMARDs or biologic/targeted synthetic DMARDs (b/tsDMARDs) than younger patients [4–8]. One study has shown that if a treat-to-target (T2T) approach is applied in EORA patients, then clinical remission and normal functioning can be achieved [14]. In contrast, most cohort studies show lower remission rates and more radiographic progression [15]. Notwithstanding, current literature is predominantly based on (retrospective) cohort studies and not clinical trials with a fixed medication protocol.

Despite the improved clinical outcomes, most RA patients still report relevant disease symptoms even in the absence of inflammation. This residual disease activity manifests itself in persistent fatigue, pain and morning stiffness, and most often has a significant impact on RA patients’ lives [16]. Therefore, nowadays a dual T2T management approach is recommended, in which the targets are [1] control of inflammation, measured with a composite disease activity index, and [2] control of disease impact, measured with several patient-reported outcomes (PROs) [17]. The International Consortium for Health Outcomes Measurement (ICHOM) agreed on the most relevant PRO domains for patients with inflammatory arthritis. These domains are pain, fatigue, activity limitation, overall emotional and physical health impact, work/school/housework ability and productivity [18].

Pavlov-Dolijanovic *et al.* already demonstrated that EORA patients have more functional impairment and experience more fatigue and depressive symptoms compared with YORA and MORA patients [10]. However, the aforementioned study had several limitations, of which the lack of a fixed medication protocol was the most important one. In addition, no or limited data are available on the other ICHOM PRO domains in the current literature.

Therefore, our aim is to investigate whether clinical outcomes (disease activity, bDMARD usage and radiologic progression) as well as ICHOM recommended PROs differ over 2 years of follow-up between YORA, MORA and EORA patients who were included in a clinical trial with a T2T management approach and a fixed medication protocol.

## Methods

### Patients

Data from the treatment in the Rotterdam Early Arthritis cohort (tREACH) trial were used. The tREACH trial was a multicentre, stratified, single-blinded randomized controlled trial with a T2T management approach and a fixed medication protocol. Patients with arthritis in one or more joint and a symptom duration of <1 year were eligible. Exclusion criteria, among others, were abnormal liver function tests, defined as aspartate aminotransferase or alanine aminotransferase >2× the upper limit of normal, or creatinine levels >150 µmol/l. The study was approved by the medical ethics committee at each participating hospital (MEC-2006–252). Written informed consent was obtained before inclusion according to the Declaration of Helsinki. A detailed description of the tREACH trial can be found elsewhere [19].

For this study, we selected all tREACH patients who fulfilled the 1987 and/or 2010 classification criteria for RA (*n* = 425) [20, 21]. These RA patients were divided into three age groups, namely:

- YORA patients, including RA patients with an age at inclusion <45 years.
- MORA patients, including patients with an age at inclusion between 45 and 65 years.
- EORA patients, including RA patients with an age at inclusion >65 years.

### Study design

In the original tREACH trial, patients were randomized into one of the following initial treatment strategies: (i) initial triple DMARD therapy (iTDT; MTX, SSZ and HCQ) with GC bridging therapy orally or i.m.; (ii) MTX with or without oral GCs (iMTX); (iii) initial HCQ monotherapy (iHCQ); or (iv) no DMARDs, initial GC once i.m. or an oral tapering scheme, or initial NSAID monotherapy.

Disease activity was assessed by a research nurse at each visit. If the target of low disease activity [disease activity score using 44 joints (DAS44) ≤2.4] was not reached, treatment was intensified. Treatment intensifications occurred in the following order: triple DMARD therapy; MTX + etanercept; MTX + adalimumab; and MTX + abatacept. Medication was tapered if the DAS44 was <1.6 at two consecutive visits. Medication was gradually discontinued, except for HCQ and NSAID, which were immediately stopped. If a flare (DAS44 > 2.4) occurred during tapering, full treatment was restarted, depending on the stage in the protocol.

### Data collection

Patients were assessed every 3 months. Clinical outcomes included DAS44, bDMARD usage and radiographic progression. The collected PROs included pain, fatigue, functional ability, quality of life, possible depression or anxiety disorder and health-related quality of life (HRQoL). At baseline, we also assessed the type and number of comorbidities, which was self-reported by the patients. Comorbidities were categorized into the following groups: diabetes, cardiovascular events, hypertension, malignancy, psychiatric disorder and others.

Disease activity was assessed with the DAS44. The DAS44 is a pooled index that incorporates a graded 53-joint count for tenderness (Ritchie Articular Index), a 44-joint count for swelling, ESR and general health [visual analogue scale (VAS) 0–100 mm] [22]. Medication data, including bDMARD and GC use, consisted of all prescribed medication for RA, which was obtained from patients’ health records. Radiographic progression was assessed with the modified Total Sharp Score (mTSS) at baseline and after 6 months, 1 and 2 years of follow-up [23]. A higher mTSS represents more radiographic progression. Radiographs were chronologically scored by two out of three qualified assessors, who were blinded for treatment allocation [24]. The weighted overall κ was 0.67 with 99% agreement.

A 0–10 Numeric Rating Scale (NRS) was used to assess pain, with lower scores indicating less pain. Fatigue was assessed with a VAS, where lower scores correspond to less fatigue. Functional ability was measured with the Health Assessment Questionnaire-Disability Index (HAQ-DI) [25]. The total score ranges from 0–3 and higher scores represent more functional impairment. Quality of life was measured with the European Quality of Life 5-Dimensions 3-Levels (EQ-5D-3L) questionnaire [26]. The EQ-5D-3L provides patients’ health status compared with the general Dutch population by assessing five health dimensions [27]. Scores range from below 0 to 1: 0 equals death, and 1 equals perfect health. Of note, the above-mentioned PROs are also recommended by the ICHOM standard set for patients with inflammatory arthritis [28].

Having a possible depression or anxiety disorder was measured with the Hospital Anxiety and Depression Scale (HADS) [29]. The HADS consists of a depression (HADS-D) and an anxiety subscale (HADS-A). Scores range from 0 to 21 per subscale and a score ≥8 is indicative of a possible depression or anxiety disorder [30].

HRQoL was measured with the 36-item Short Form Health Survey (SF-36) [31]. The SF-36 covers eight domains, namely physical functioning, bodily pain, role limitations due to physical health problems, role limitations due to personal or emotional problems, general mental health, social functioning, energy/fatigue and general health perceptions. The summed scores per domain are transformed to a 0–100 scale, where a higher score reflects a better HRQoL. A Physical Component Summary (PCS) and Mental Component Summary (MCS) score were also calculated from these domains.

### Statistical analysis

Differences in baseline characteristics and at fixed timepoints, namely after 12 and 24 months of follow-up, between age groups were evaluated with an analysis of variance, Kruskal–Wallis test or χ^2^ test, when appropriate.

For our analysis over time, we used a multilevel mixed-effects linear regression model with correlated random intercepts and slopes for continuous outcomes. For binary outcomes (i.e. bDMARD use, remission and possible depression or anxiety disorder), a multilevel mixed-effects logistic regression model with an unstructured covariance matrix was used. The models included age groups (YORA, MORA, EORA), time, sex, number of comorbidities, symptom duration, baseline DAS44, baseline mTSS and ACPA positivity as covariates.

If a PRO significantly differed between age groups, then this difference was compared with the minimal clinically important difference (MCID). The MCID can be used as a criterion to determine whether significant differences are also clinically relevant. The MCID values for pain, fatigue, HAQ-DI, EQ-5D-3L and SF-36 were ≥1, ≥10, ≥0.22, ≥0.04 and ≥3–5, respectively [32–35].

Dropout rates for the YORA, MORA and EORA after 1 year of follow-up were 7%, 3% and 6%, which increased to 18%, 13% and 12% after 2 years of follow-up, respectively. Missing values were not imputed, but they were handled with our mixed models.

To validate our results, we performed three sensitivity analyses. First, a complete case analysis was performed to strengthen confidence that missingness was at random. Complete cases were defined as patients who had a DAS44 at each timepoint. Secondly, a cut-off of 70 years was used for the definition of EORA, because of the changes in the life expectancy one can argue that 70 might be a better cut-off for the definition of elderly [36]. Lastly, a subgroup analysis that only included autoantibody positive RA patients, defined as RF or ACPA positive, was conducted, because these patients have the worst prognosis.

A *P*-value <0.05 was considered statistically significant. Analyses were performed in STATA v18.

## Results

### Patient

A total of 587 patients were included in the original tREACH trial, of whom 425 fulfilled the 1987 and/or 2010 classification criteria for RA (Supplementary Fig. S1). At diagnosis, 119 (28%) patients were younger than 45 years (YORA), 208 (49%) patients were between 45 and 65 years (MORA), and 98 (23%) patients were older than 65 years (EORA).

EORA patients were more often male and had more comorbidities compared with their younger counterparts. They also had more erosions, more swollen joints, and higher ESR and CRP levels. However, EORA patients had fewer tender joints than the other two age groups. The DAS44, therefore, did not significantly differ between age groups at baseline (Table 1).

**Table 1.** Baseline characteristics stratified for age group

	<45 years (*n* = 119)	45–65 years (*n* = 208)	>65 years (*n* = 98)	*P*-value
Demographic characteristics				
Sex (female), *n* (%)	90 (76)	147 (71)	49 (50)	**<0.001**
Age (years), mean (s.d.)	36.5 (7.1)	55.1 (5.5)	72.6 (4.9)	**<0.001**
Comorbidities, *n* (%)				
No comorbidity	74 (64)	89 (43)	23 (24)	**<0.001**
≥2 comorbidities	41 (34)	91 (44)	56 (57)	**0.004**
Type of comorbidities				
Diabetes	1 (1)	13 (8)	10 (15)	**0.006**
Cardiovascular events	5 (6)	30 (18)	30 (39)	**<0.001**
Hypertension	4 (5)	39 (25)	34 (43)	**<0.001**
Malignancy	1 (1)	5 (3)	7 (11)	**0.009**
Psychiatric disorder	14 (15)	29 (18)	7 (11)	0.41
Other	27 (26)	62 (34)	37 (43)	0.06
Disease characteristics				
Symptom duration (months), median (IQR)	4.8 (2.9–7.0)	5.2 (3.1–7.3)	4.1 (2.8–6.2)	**0.04**
ACPA positive, *n* (%)	67 (56)	106 (51)	47 (48)	0.4
RF positive, *n* (%)	63 (53)	105 (50)	48 (49)	0.8
mTSS, median (IQR)	0 (0–0.5)	0.5 (0–1.5)	1.2 (0–4)	**<0.001**
JSN, median (IQR)	0 (0–0)	0 (0–1)	0.5 (0–2.5)	**<0.001**
ES, median (IQR)	0 (0–0)	0 (0–0.5)	0 (0–1)	**<0.001**
Disease activity				
DAS44, mean (s.d.)	3.2 (0.9)	3.3 (0.9)	3.5 (1.0)	0.1
SJC44, median (IQR)	6 (3–9)	7 (4–12)	10 (6–15)	**<0.001**
TJC53, median (IQR)	10 (5–15)	10 (5–15)	8 (4–12)	**0.04**
ESR (mm/h), median (IQR)	18 (10–28)	18 (11–36)	33 (17–50)	**<0.001**
GH (VAS), median (IQR)	53 (34–70)	53 (30–67)	49 (29–68)	0.26
CRP (mg/L), median (IQR)	7 (3–15)	6 (3–17)	16 (6–42)	**<0.001**
Initial treatment, *n* (%)				
iTDT	55 (46)	78 (38)	40 (41)	0.3
iMTX	31 (26)	79 (38)	34 (35)	0.09
iHCQ	19 (16)	25 (12)	13 (13)	0.6
No DMARDS	14 (12)	26 (13)	11 (11)	0.95
MTX dose at T3, median (IQR)	25 (25–25)	25 (25–25)	25 (25–25)	0.37

*P* < 0.05 was considered significant and is shown in bold. DAS44: DAS44 with four items [SJC44, TJC53, ESR, GH (VAS 0–100 mm)]; ES: Erosion Score; GH: general health; iTDT: initial triple disease-modifying antirheumatic drug therapy (MTX, SSZ and HCQ) with glucocorticoid bridging therapy oral or intramuscularly; iMTX, initial MTX with or without an oral glucocorticoid tapering scheme; iHCQ: initial HCQ monotherapy; IQR: interquartile range; JSN: Joint Space Narrowing; mTSS: modified Total Sharp Score; no DMARDs: initial glucocorticoid i.m. or oral tapering scheme or initial NSAID monotherapy; SJC: swollen joint count; TJC: tender joint count; VAS: visual analogue scale.

### Clinical outcomes

**Figure 1.**
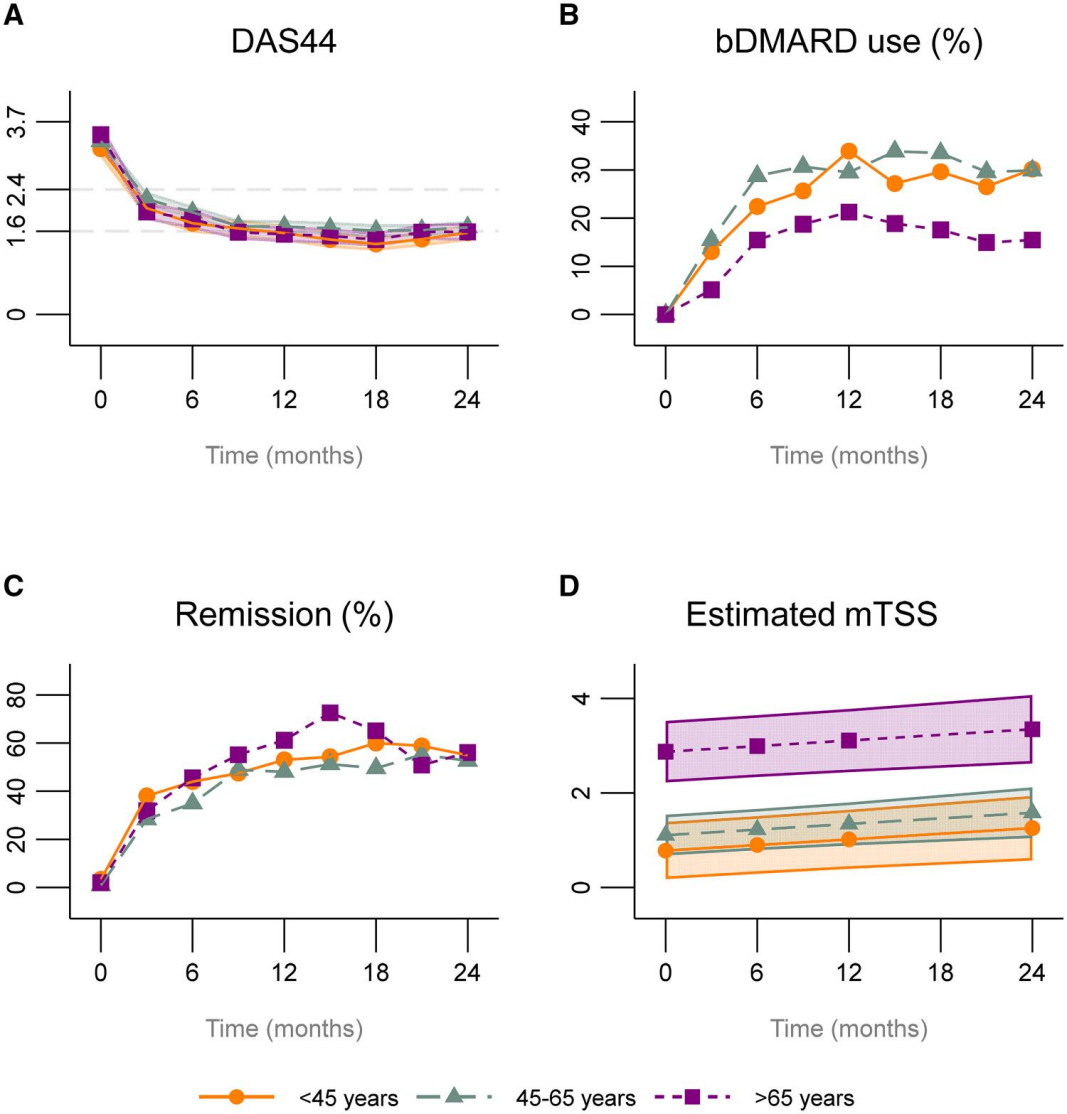
Clinical outcomes during the 2-year follow-up period stratified for age group. (**A**) Mean DAS44 score; (**B**) proportion of patients using a bDMARD; (**C**) proportion of patients in DAS44 remission; (**D**) estimated mean mTSS (114 patients <45 years, 205 patients between 45 and 65 years, and 96 patients >65 years), adjusted for sex, number of comorbidities, symptom duration, time, ACPA positivity and baseline DAS44. DAS44: DAS44 with four items (swollen joint count 44, tender joint count 53, ESR), general health (visual analogue scale 0–100 mm); mTSS: modified Total Sharp Score; bDMARD: biologic DMARD

No differences were seen in DAS44 over time between the age groups (Table 2, Fig. 1A). Also, no differences in remission rates were observed (Table 2, Fig. 1C). Although not significant, the odds ratios (95% CI) of using a bDMARD over time were 1.4 (0.4–4.6) and 1.9 (0.7–5.3) in YORA and MORA patients compared with EORA patients, respectively (Table 2, Fig. 1B). However, after 2 years of follow-up, bDMARD usage among EORA patients (15%) was significantly lower than YORA patients (30%) and MORA patients (30%) (Table 3). Even though the tREACH trial had a fixed medication protocol, not every rheumatologist always adhered to the protocol. Consequently, we examined whether there was a difference in (chronic) GC usage between the age groups and discovered no differences (Supplementary Fig. S2).

**Table 2.** Clinical and patient-reported outcomes over the 2-year follow-up period stratified for age group

	<45 years (*n* = 119)	45-65 years (*n* = 208)	>65 years (*n* = 98)
Clinical outcomes			
DAS44, mean diff (95% CI)	–0.1 (–0.3 to 0.1)	0 (–0.2 to 0.2)	Ref
bDMARD use, OR (95% CI)	1.4 (0.4 to 4.6)	1.9 (0.7 to 5.3)	Ref
Remission, OR (95% CI)	0.9 (0.5 to 1.6)	0.8 (0.5 to 1.2)	Ref
mTSS, mean diff (95% CI)	–2.1 (–3 to –1.2)***	–1.8 (–2.5 to –1)***	Ref
JSN	–1.1 (–1.7 to –0.5)**	–0.8 (–1.3 to –0.3)**	Ref
ES	–0.8 (–1.4 to –0.3)**	–0.7 (–1.2 to –0.3)**	Ref
Patient-reported outcomes			
Pain, mean diff (95% CI)	**1 (0.5 to 1.6)** ***	0.6 (0.1 to 1)*	Ref
Fatigue, mean diff (95% CI)	**17.3 (11.3 to 23.4)** ***	5.8 (0.7 to 10.9)*	Ref
Functional ability (HAQ-DI), mean diff (95% CI)	0 (–0.14 to 0.14)	–0.06 (–0.18 to 0.06)	Ref
Quality of life (EQ-5D-3L), mean diff (95% CI)	–0.03 (–0.07 to 0.01)	–0.03 (–0.06 to 0.01)	Ref
Depression (HADS), OR (95% CI)	4.2 (1.3 to 13.6)*	2.4 (0.9 to 6.5)	Ref
Anxiety (HADS), OR (95% CI)	8.4 (2.1 to 33.1)**	2.9 (0.9 to 9)	Ref
PCS (SF-36), mean diff (95% CI)	0.1 (–2.3 to 2.4)	1.1 (–1 to 3.1)	Ref
MCS (SF-36), mean diff (95% CI)	–2.7 (–5.2 to –0.3)*	–1.8 (–3.8 to 0.3)	Ref

The differences that exceeds the MCID and are, thus, clinically relevant are shown in bold.

All analyses were adjusted for sex, number of comorbidities, symptom duration, time, ACPA positivity and baseline DAS44 (except for DAS44) and mTSS (except for mTSS).

* *P* < 0.05,

** *P* < 0.01,

*** *P* < 0.001. bDMARD: biologic DMARD; DAS44: DAS44 with four items [swollen joint count 44, tender joint count 53, ESR, general health (VAS 0–100 mm)]; diff, difference; EQ-5D-3L: European Quality of life 5-Dimensions 3-Levels; ES: Erosion Score; HADS: Hospital Anxiety and Depression Scale; HAQ-DI: HAQ-Disability Index; JSN: Joint Space Narrowing; MCS: Mental Component Scale; MCID: minimal clinically important difference; mTSS: modified Total Sharp Score; OR: odds ratio; PCS: Physical Component Scale; Ref: reference group; SF-36: 36-item Short Form Health Survey; VAS: visual analogue scale.

**Table 3.** Outcomes per timepoint stratified for age group

	Time	<45 years (*n* = 119)	45-65 years (*n* = 208)	>65 years (*n* = 98)
Clinical outcomes				
DAS44, mean (s.d.)	T12	1.6 (0.9)	1.7 (0.9)	1.5 (0.8)
	T24	1.6 (0.9)	1.7 (0.9)	1.6 (0.9)
LDA (DAS44 ≤ 2.4), *n* (%)	T12	78 (80)	150 (82)	71 (84)
	T24	67 (84)	124 (83)	56 (85)
Remission (DAS44 < 1.6), *n* (%)	T12	52 (53)	88 (48)	52 (61)
	T24	44 (55)	79 (53)	37 (56)
bDMARD use, *n* (%)	T12	38 (34)	59 (30)	20 (21)
	T24*	32 (30)	55 (30)	13 (15)
mTSS, median (IQR)	T12*	0 (0–1)	0.5 (0–2)	1 (0–4)
	T24	0 (0–0)	0 (0–1)	0 (0–1.5)
Patient-reported outcomes				
Pain (0–10 NRS), median (IQR)	T12	2 (1–6)	2 (1–5)	1 (0–3)
	T24	2 (0–5)	2 (0–5)	1.5 (1–5)
Fatigue (VAS), median (IQR)	T12*	**54 (25–73)**	**37 (19–68)**	**30 (11–56)**
	T24	44 (11–70)	35 (20–65)	28 (17–52)
Functional ability (HAQ-DI), median (IQR)	T12	0.5 (0–1.12)	0.38 (0–1)	0.5 (0.12–1.12)
	T24	0.5 (0–1)	0.62 (0–1)	0.5 (0.12–1.12)
Quality of life (EQ-5D-3L), mean (s.d.)	T12	0.78 (0.15)	0.76 (0.17)	0.78 (0.15)
	T24	0.81 (0.13)	0.77 (0.18)	0.78 (0.17)
Depression (HADS-D ≥8), *n* (%)	T12	9 (11)	27 (16)	9 (12)
	T24	5 (8)	16 (12)	8 (14)
Anxiety (HADS-A ≥8), *n* (%)	T12	16 (19)	33 (19)	10 (13)
	T24	9 (14)	23 (17)	6 (11)
PCS (SF-36), mean (s.d.)	T12	43 (11)	43 (10)	42 (11)
	T24	44 (10)	44 (10)	43 (11)
MCS (SF-36), mean (s.d.)	T12	52 (9)	53 (9)	53 (10)
	T24	53 (8)	52 (10)	55 (8)

The differences that exceed the MCID and are, thus, clinically relevant are shown in bold. The MCID values for pain, fatigue, HAQ-DI, EQ-5D-3L, and SF-36 were ≥1, ≥10, ≥0.22, ≥0.04 and ≥3–5, respectively.

* *P* < 0.05 was considered significant. bDMARD: biologic DMARD; DAS44: DAS44 with four items [swollen joint count 44, tender joint count 53, ESR, general health (VAS of 100 mm)]; EQ-5D-3L: European Quality of life 5-Dimensions 3-Levels; HADS: Hospital Anxiety and Depression Scale; HAQ-DI: HAQ-Disability Index; IQR: interquartile range; MCID: minimal clinically important difference; MCS: Mental Component Scale; mTSS: modified Total Sharp Score; NRS: Numeric Rating Scale; PCS: Physical Component Scale; SF-36: 36-item Short Form Health Survey; VAS: visual analogue scale.

At baseline, EORA patients had a higher mTSS score [median (interquartile range); 1.2 (0–4) in EORA patients compared with 0 (0–0.5) in YORA and 0 (0–1.5) in MORA patients] (Table 1). EORA patients also had significantly more radiographic progression over time, with approximately a 2-point mean difference on mTSS (Table 2, Fig. 1D). To get a better understanding of the difference in mTSS over time, we also examined the joint space narrowing and erosion score separately. Both were significantly worse in EORA patients (Table 2, Supplementary Fig. S3).

### Patient-reported outcomes

**Figure 2.**
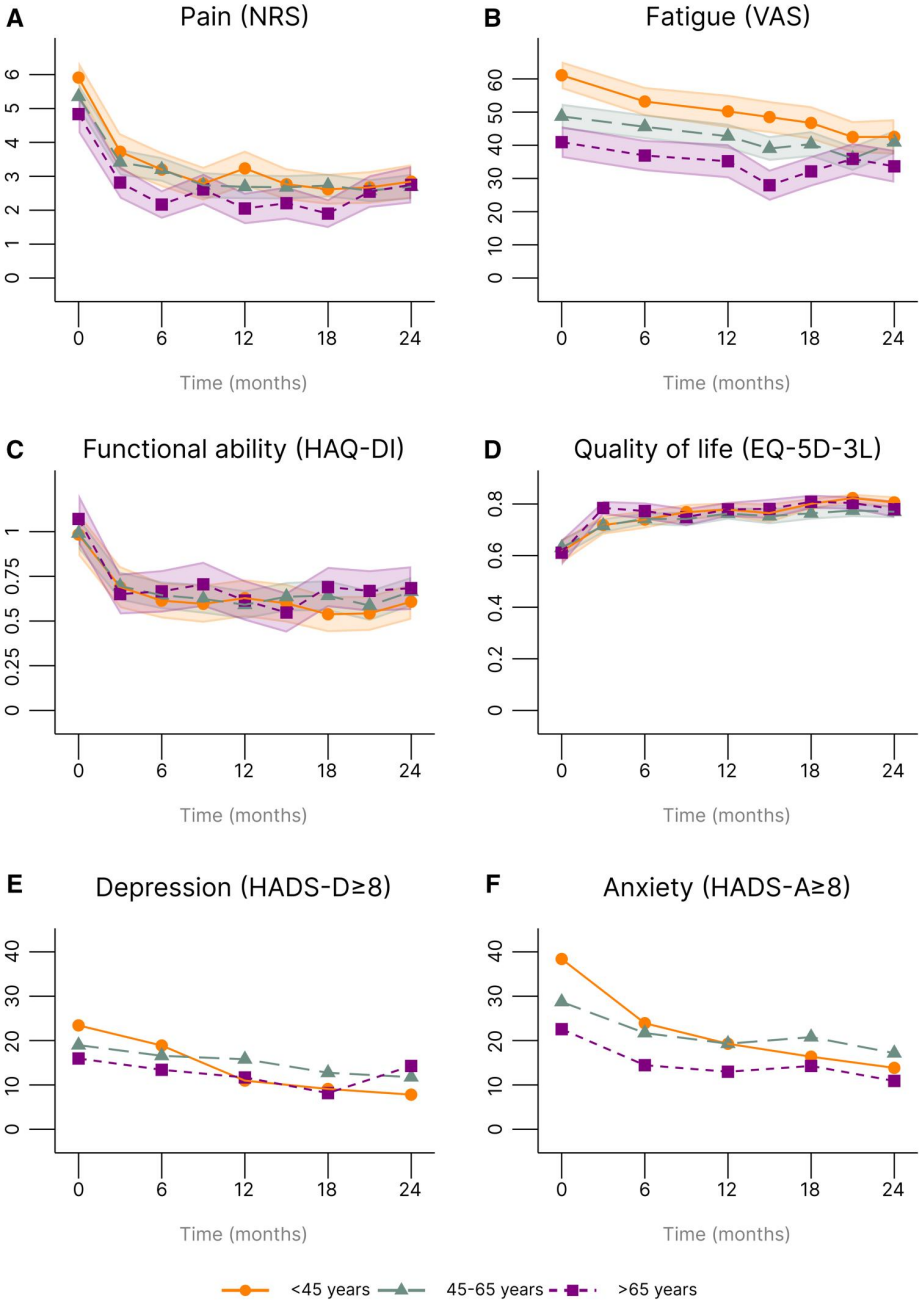
Patient-reported outcomes during the 2-year follow-up period stratified for age group. (**A**) Mean pain (0–10 NRS); (**B**) mean fatigue (VAS); (**C**) mean functional ability (HAQ-DI); (**D**) mean quality of life (EQ-5D-3L); (**E**) % of patients with a possible depression (HADS-D ≥8); and (**F**) % of patients with a possible anxiety disorder (HADS-A ≥8). EQ-5D-3L: European Quality of Life 5-Dimensions 3-Levels; HADS: Hospital Anxiety and Depression Scale; HADS-A: HADS-Anxiety score; HADS-D: HDS-Depression score; HAQ-DI: HAQ-Disability Index; NRS: Numeric Rating Scale; VAS: visual analogue scale

PROs in all age groups improved over time. EORA patients, however, experienced less pain over time compared with YORA patients [estimated mean difference (95% CI), 1 (0.5–1.6)] and MORA patients [0.6 (0.1–1)] (Table 2, Fig. 2A). The MCID (≥1) was only exceeded between EORA and YORA patients [33]. Similar results were found for fatigue [EORA (ref) *vs* YORA 17.3 (11.3–23.4) and EORA (ref) *vs* MORA 5.8 (0.7–10.9)]. The MCID (≥10) was again only exceeded for the comparison between EORA and YORA patients (Table 2, Fig. 2B) [32].

Functional ability (HAQ-DI) and quality of life (EQ-5D-3L) did not significantly differ over time between age groups (Table 2, Fig. 2C and D) [34, 35]. The odds ratio (95% CI) of having a possible depression or anxiety disorder over time was significantly higher in YORA patients compared with EORA patients [4.2 (1.3–13.6) and 2.4 (0.9–6.5), respectively] (Table 2, Fig. 2E and F).

After 24 months of follow-up, no PRO differences were observed between the age groups (Table 3).

**Figure 3.**
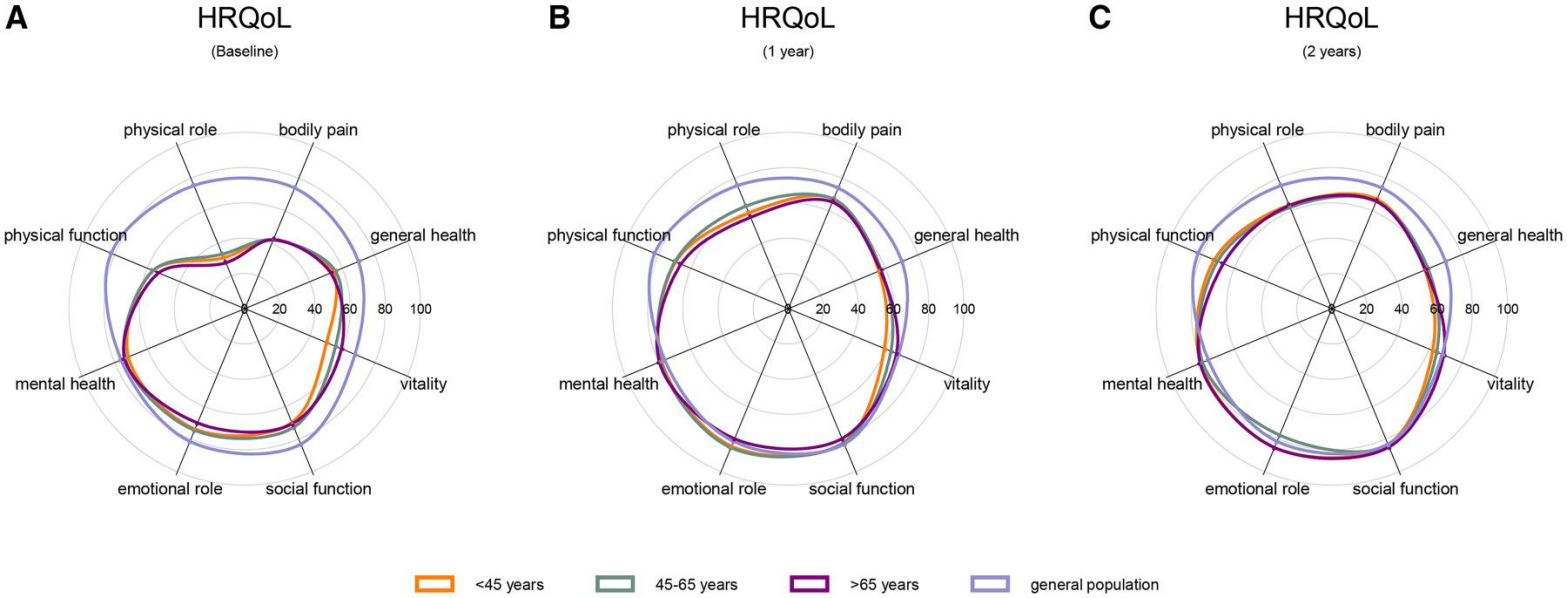
HRQoL for age groups and the general Dutch population. (**A**) Mean HRQoL scores at baseline; (**B**) after 1 year; and (**C**) after 2 years, measured with the 36-item Short Form Health Survey, stratified for age-group and compared with the general Dutch population norms. HRQoL: health-related quality of life

The eight domains of the SF-36 were also examined at baseline and after 12 and 24 months of follow-up and compared with the Dutch population norm. In all three age groups, most problems were experienced in the physical domains, which improved after treatment, but they never reached the Dutch population norm (Fig. 3). YORA patients had a worse SF-36 MCS over time [–2.7 (95% CI –5.2 to –0.3)] compared with EORA patients, but the MCID (≥3–5) was not exceeded [34]. No differences between age groups over time were found for the SF-36 PCS (Table 2).

### Sensitivity analysis

Across all three sensitivity analyses—(i) complete case analysis; (ii) EORA defined as individuals aged 70 years and older; and (iii) subgroup analysis of autoantibody positive RA patients (either RF or ACPA positive)—similar clinical outcomes and PROs were observed over time, and after 12 and 24 months of follow-up (Supplementary Tables S1–S6 and Figs S4–S12).

## Discussion

In this study with a T2T management approach and a fixed medication protocol, we investigated whether clinical outcomes as well as PROs differ over 2 years between patients with an YORA, MORA or EORA at the time of diagnosis. At diagnosis, EORA patients were more often male, had more swollen joints, more erosions, higher inflammation markers and more comorbidities compared with their younger counterparts. Disease activity at diagnosis and over time, however, did not differ between age groups. After 2 years of follow-up, bDMARD usage among EORA patients was significantly lower than YORA patients and MORA patients, while (chronic) GC usage was similar between age groups. Yet, EORA patients had more radiographic progression, but the mean difference over time was only ∼2 points on the mTSS that ranges from 0 to 448 points. Conversely, EORA patients experienced less pain and fatigue compared with YORA and MORA patients. YORA patients also had more mental complaints than EORA patients.

Current literature shows that EORA patients have worse outcomes [4]. One reason might be that rheumatologists tend not to comply with the T2T management approach, because of their advanced age and multiple comorbidities [4, 5]. Consequently, EORA patients more often receive GCs and less often DMARD combination therapies or bDMARDs [6–8]. However, the current viewpoint is mainly based on (retrospective) cohort studies and expert opinion [9–11]. Acuña-Rocha *et al.* for example, showed that despite the unfavourable prognostic factors at diagnosis, EORA patients achieve similar long-term remission rates compared with their younger counterparts if a T2T approach is applied [37]. In our study, EORA patients also had no problem adhering to the T2T management approach, even though ∼60% had two or more comorbidities. We therefore encourage to stive for a T2T management approach in EORA patients, irrespective of the number of comorbidities. Nonetheless, comorbidities should be considered when choosing DMARD treatment in shared decision-making with the patients as well as the overall risk–benefit balance.

More importantly, fewer EORA patients required bDMARDs to reach the same treatment goal without using more GCs. The need for less intensive DMARD therapy might be due to aging of the immune system, also known as immunosenescence [38]. Immunosenescence results in an altered immune response, which manifests itself in a higher susceptibility to infections, poorer vaccination efficacy and a higher incidence of inflammatory diseases, including cardiovascular disease, and malignancies [39]. Changes in the immune system could lead to a reduced need for intensive DMARD therapy in EORA patients [37].

Still, our EORA patients had more erosions compared with their younger counterparts. This aligns with the findings from the NOAR registry, which showed that a higher age at diagnosis was associated with more erosions [40]. However, it is important to note that erosions may also develop in healthy individuals, particularly in those over the age of 50 years [41]. Additionally, our EORA patients had more radiographic progression over time, which is associated with more functional impairment. Yet, we did not find any differences in functioning between the age groups [15]. A possible explanation might be that the mean difference in radiographic progression (∼2 mTSS points) is too small to cause a difference in functioning. Another explanation might be that functional ability was measured with the HAQ-DI, which only correlates with the Sharp van der Heijde score if there is enough joint damage [42, 43].

Besides control of inflammation, the disease impact should also be addressed; therefore, nowadays a dual T2T management approach is recommended [17]. The impact of disease can be measured with PROs. In our study, functional ability and quality of life were similar between the age groups, while EORA patients experienced less pain, fatigue and mental complaints compared with their younger counterparts. Unfortunately, previous literature showed inconsistent findings [10, 37, 44]. Nevertheless, if a T2T management approach was applied—similar to our study—then the results aligned with our findings [37]. The difference in experienced pain, fatigue and mental complaints between EORA patients and their younger counterparts can hypothetically be explained by differences in coping skills and generational beliefs, whereas social isolation and multimorbidity can also aggravate PROs [45–47].

The strengths of our study are the trial design and the broad clinical outcomes as well as PROs that cover multiple ICHOM domains. However, this made multiple testing inevitable. Therefore, significant PROs were compared with the minimal clinically important differences, which made our results more robust. Secondly, a healthy volunteer effect is a potential concern when patients are enrolled in a trial, especially if they have a higher age. In our study, EORA covered a quarter of the included patients, while its prevalence at diagnosis is around a third in the general RA population. Nonetheless, baseline characteristics of our EORA patients were comparable to previous literature. Another concern could be the presence of an age bias; however, our design, which included a T2T management approach and a fixed medication protocol, made this possibility—in our opinion—unlikely. Finally, prior research showed conflicting results about the prevalence of autoantibodies in YORA, MORA and EORA patients. For this reason, we conducted a subgroup analysis focusing on autoantibody positive patients, which showed similar results. We, therefore, think that our results are generalizable to RA patients who are managed with a T2T approach, irrespective of their age and number of comorbidities. Still, future research is necessary to confirm our findings, especially the finding that EORA patients need less intensive therapy and might even have better long-term clinical outcomes.

In conclusion, despite unfavourable prognostic factors at diagnosis, EORA patients have similar outcomes compared with their younger counterparts if a T2T management approach is applied. Interestingly, fewer EORA patients needed bDMARDs to reach the same treatment target compared with their younger counterparts, while (chronic) GC usage was similar across all age groups. We, therefore, recommend that one should strive to apply a T2T management approach in EORA patients, irrespective of the number of comorbidities, but the choice of DMARD treatment should be tailored to the individual patient.

## Supplementary Material

keaf432_Supplementary_Data

## Data Availability

Data are available from the corresponding author upon reasonable request.
