# Supplementary material for: Eye-openers for advanced management of elderly-onset rheumatoid arthritis
Source: Rheumatology (Oxford). 2025 Aug 9;64(12):6241–9. doi: 10.1093/rheumatology/keaf432 (PMC12671882; doi:10.1093/rheumatology/keaf432)
Supplement: keaf432_Supplementary_Data [file keaf432_supplementary_data.docx]

**SUPPLEMENTARY MATERIALS**


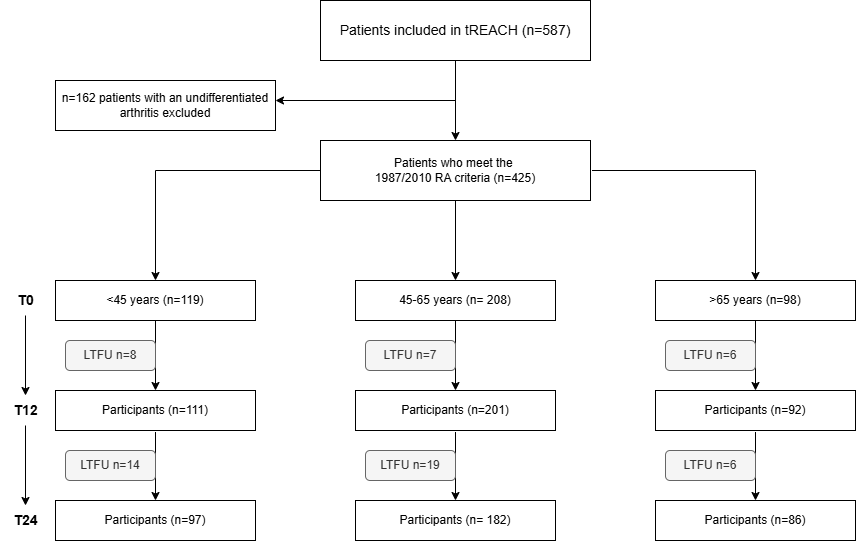


**Supplementary Figure S1.** Flowchart.

*Abbreviations: LTFU, loss to follow-up; RA, Rheumatoid Arthritis; and tREACH, treatment in the Rotterdam Early Arthritis CoHort.*

**
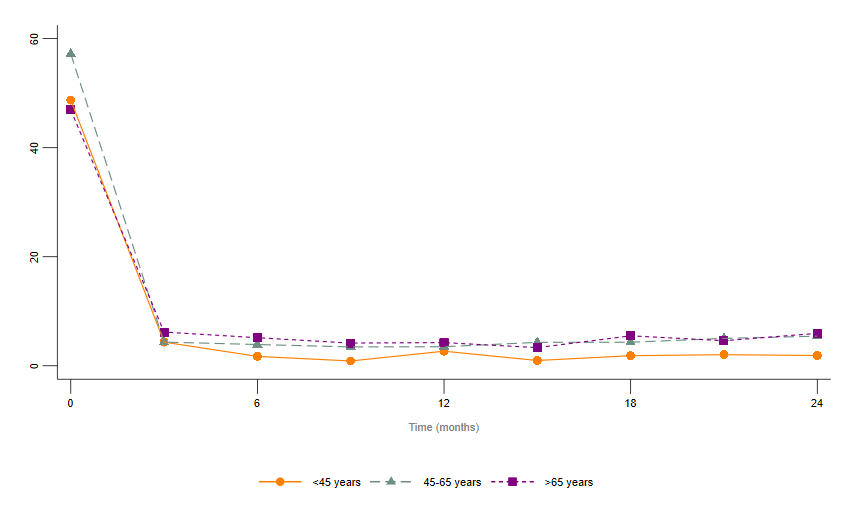
**

**Supplementary Figure S2.** Proportion of patients (chronically) using glucocorticoids, stratified for age-group.

**
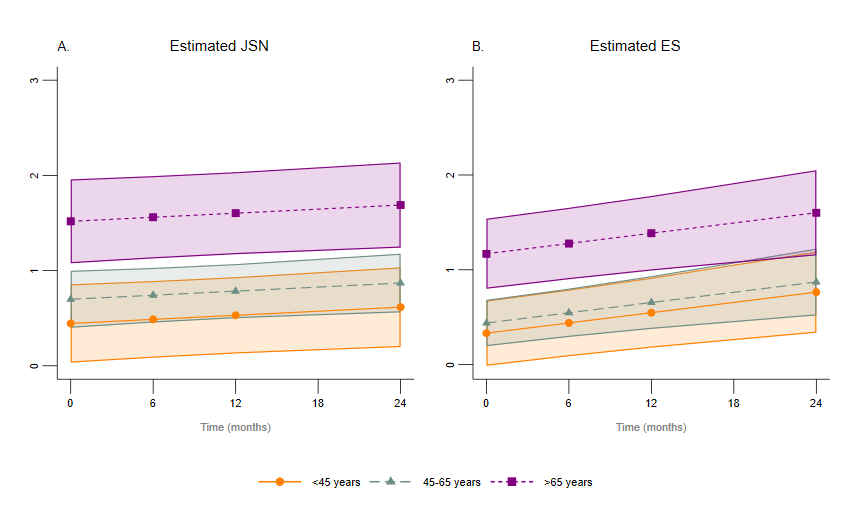
**

**Supplementary Figure S3.** Estimated joint space narrowing (JSN) and erosion score (ES) stratified for age-group. Components of the mTSS score, including **(A)** JSN and **(B)** ES, for 114 RA patients under 45 years, 205 RA patients between 45 and 65 years, and 96 RA patients over 65 years. Data are adjusted for sex, number of comorbidities, symptom duration, time, ACPA positivity, and baseline DAS44.

*Abbreviations: ACPA, anti-citrullinated protein antibody; DAS44, disease activity score 44 with 4 items (swollen joint count 44, tender joint count 53, erythrocyte sedimentation rate, general health (Visual Analogue Scale 0-100mm)); ES, erosion score; JSN, joint space narrowing; mTSS, modified Total Sharp Score.*

**Supplementary Table S1. Clinical and patient-reported outcomes for complete cases during the 2-year follow-up period stratified for age-group.**

|  | **<45 years**  **(n=55)** | **45-65 years**  **(n=94)** | **>65 years**  **(n=53)** |
| --- | --- | --- | --- |
| **Clinical outcomes** |  | |  |
| - DAS44*, mean diff (95% CI)* | -0.1 (-0.4 to 0.1) | 0.1 (-0.1 to 0.3) | Ref |
| - bDMARD use*, OR (95% CI)* | 0.8 (0.2 to 4) | 1.7 (0.5 to 6.2) | Ref |
| - Remission*, OR (95% CI)* | 1 (0.5 to 1.9) | 0.7 (0.4 to 1.2) | Ref |
| - mTSS*, mean diff (95% CI)* | -2.4 (-3.6 to -1.2)^3^ | -1.7 (-2.7 to -0.7)^2^ | Ref |
| - JSN | -1.1 (-2 to -0.2)^1^ | -0.7 (-1.5 to 0.2) | Ref |
| - ES | -1.2 (-1.8 to -0.7)^3^ | -1 (-1.5 to -0.6)^3^ | Ref |
| **Patient-reported outcomes** |  | |  |
| - Pain (NRS)*, mean diff (95% CI)* | **1.1 (0.5 to 1.8)^2^** | 0.8 (0.2 to 1.3)^2^ | Ref |
| - Fatigue (VAS), *mean diff (95% CI)* | **18.5 (10.9 to 26.2)^3^** | 8.3 (1.8 to 14.9)^1^ | Ref |
| - Functional ability (HAQ-DI)*, mean diff (95% CI)* | 0.09 (-0.08 to 0.26) | 0.01 (-0.13 to 0.15) | Ref |
| - Quality of life (EQ-5D-3L), *mean diff (95% CI)* | -0.03 (-0.08 to 0.02) | -0.03 (-0.07 to 0.02) | Ref |
| - Depression (HADS)*, OR (95% CI)* | 2.3 (0.6 to 9.2) | 1.7 (0.5 to 5.3) | Ref |
| - Anxiety (HADS)*, OR (95% CI)* | 4.4 (0.7 to 26.2) | 1.7 (0.4 to 7.5) | Ref |
| - PCS (SF-36)*, mean diff (95% CI)* | -1.4 (-4.5 to 1.7) | -1.3 (-3.9 to 1.3) | Ref |
| - MCS (SF-36)*, mean diff (95% CI)* | -1.1 (-4.1 to 2) | -0.2 (-2.7 to 2.4) | Ref |

^1^p < 0.05, ^2^p < 0.01, ^3^p < 0.001

The differences that exceed the MCID and are, thus, clinically relevant are shown in bold.

All analyses were adjusted for sex, number of comorbidities, symptom duration, time, ACPA positivity, and baseline DAS44 (except for DAS44) and mTSS (except for mTSS).

*Abbreviations: ACPA, anti-citrullinated protein antibody; bDMARD, biologic disease-modifying antirheumatic drug; DAS44, disease activity score 44 with 4 items (swollen joint count 44, tender joint count 53, erythrocyte sedimentation rate (ESR), general health (Visual Analogue Scale (VAS) 0-100 mm)); diff, difference; EQ-5D-3L, European Quality of life 5-Dimensions 3-Levels; ES, Erosion Score; HADS, Hospital Anxiety and Depression Scale; HAQ-DI, Health Assessment Questionnaire–Disability Index; JSN, Joint Space Narrowing; MCS, Mental Component Scale; MCID, minimal clinically important difference; mTSS, modified Total Sharp Score; OR, odds ratio; PCS, Physical Component Scale; Ref, reference group; SF-36, 36-item Short Form Health Survey; and VAS, Visual Analogue Scale.*

**Supplementary Table S2. Outcomes per timepoint for complete cases stratified for age-group.**

|  | **Time** | **<45 years**  (n= 55) | **45-65 years**  (n= 94) | **>65 years** (n = 53) |
| --- | --- | --- | --- | --- |
| **Clinical outcomes** | | | | |
| **DAS44**, *mean (sd)* | T12 | 1.4 (0.8) | 1.6 (0.9) | 1.4 (0.7) |
|  | T24 | 1.5 (0.8) | 1.7 (0.9) | 1.5 (0.8) |
| **LDA,** *n (%)* | T12 | 47 (85) | 78 (83) | 47 (89) |
| (DAS44 ≤ 2.4) | T24 | 47 (85) | 79 (84) | 48 (91) |
| **Remission,** *n (%)* | T12 | 31 (56) | 48 (51) | 38 (72) |
| (DAS44 < 1.6) | T24 | 31 (56) | 49 (52) | 33 (62) |
| **bDMARD use,** *n (%)* | T12 | 19 (35) | 33 (35) | 9 (17) |
|  | T24* | 16 (30) | 33 (36) | 7 (14) |
| **mTSS,** *median (IQR)* | T12* | 0.3 (0 - 1) | 0.8 (0 - 2) | 2 (1 - 4.5) |
|  | T24* | 0 (0 - 0.5) | 0 (0 - 2) | 1 (0 – 3.8) |
| **PROs** | | | | |
| **Pain (NRS),** *median (IQR)*  *MCID ≥1* | T12 | 2 (1 - 5) | 2 (1 - 5) | 1 (0 - 3) |
|  | T24 | 2 (1 - 6) | 1 (0 - 5) | 1 (0 - 4) |
| **Fatigue (VAS),** *median (IQR)*  *MCID ≥10* | T12* | **54 (29 - 73)** | **37 (20 - 68)** | **31 (11 - 54)** |
|  | T24 | 48 (20 - 74) | 35 (22 - 65) | 23 (16 - 50) |
| **Functional ability (HAQ-DI),** *median (IQR)*  *MCID ≥0.22* | T12 | 0.56 (0 – 1.06) | 0.38 (0 - 1) | 0.5 (0 - 1) |
|  | T24 | 0.62 (0 - 1.25) | 0.62 (0 - 1.12) | 0.5 (0 – 1.06) |
| **Quality of life (EQ-5D-3L),** *mean (sd)*  *MCID ≥0.04* | T12 | 0.77 (0.17) | 0.77 (0.14) | 0.78 (0.14) |
|  | T24 | 0.79 (0.15) | 0.76 (0.19) | 0.78 (0.17) |
| **Depression (HADS-D≥8),** *n (%)* | T12 | 4 (8) | 13 (14) | 6 (12) |
|  | T24 | 2 (5) | 10 (13) | 5 (12) |
| **Anxiety (HADS-A≥8),** *n (%)* | T12 | 8 (17) | 14 (16) | 6 (12) |
|  | T24 | 6 (14) | 13 (17) | 4 (10) |
| **PCS (SF-36),** *mean (sd)*  *MCID ≥3-5* | T12 | 43 (11) | 42 (10) | 42 (11) |
|  | T24 | 44 (9) | 43 (11) | 44 (11) |
| **MCS (SF-36),** *mean (sd)*  *MCID ≥3-5* | T12 | 52 (10) | 53 (9) | 53 (10) |
|  | T24 | 54 (8) | 52 (10) | 56 (8) |

*p < 0.05 was considered significant.

The differences that exceed the MCID and are, thus, clinically relevant are shown in bold.

*Abbreviations: bDMARD, biologic disease-modifying antirheumatic drug; DAS44, disease activity score 44 with 4 items (swollen joint count 44, tender joint count 53, erythrocyte sedimentation rate, general health (VAS of 100 mm)); EQ-5D-3L, European Quality of life 5-Dimensions 3-Levels; HADS, Hospital Anxiety and Depression Scale; HAQ-DI, Health Assessment Questionnaire–Disability Index; IQR, interquartile range; MCID, minimal clinically important difference; MCS, Mental Component Scale; mTSS, modified Total Sharp Score; NRS, Numeric Rating Scale; PCS, Physical Component Scale; sd, standard deviation; SF-36, 36-item Short Form Health Survey; and VAS, Visual Analogue Scale.*

**Supplementary Figure S4.** Clinical outcomes for complete cases during the 2-year follow-up period stratified for age-group. **(A)** Mean DAS44 score; **(B)** Proportion of patients using a bDMARD; **(C)** Proportion of patients in DAS44 remission; **(D)** Estimated mean mTSS (53 patients under 45 years, 93 patients between 45 and 65 years, and 52 patients older than 65 years), adjusted for sex, number of comorbidities, symptom duration, time, ACPA positivity, and baseline DAS44.

*Abbreviations: ACPA, anti-citrullinated protein antibody; DAS44, disease activity score 44 with 4 items (swollen joint count 44, tender joint count 53, erythrocyte sedimentation rate (ESR), general health (Visual Analogue Scale (VAS) 0-100 mm)); and mTSS, modified Total Sharp Score.*

**Supplementary Figure S5.** Patient-reported outcomes for complete cases during the 2-year follow-up period stratified for age-group. **(A)** Mean pain (NRS); **(B)** Mean fatigue (VAS); **(C)** Mean functional ability (HAQ-DI); **(D)** Mean quality of life (EQ-5D-3L). **(E)** % of patients with a possible depression (HADS-D ≥8); and **(F)** % of patients with a possible anxiety disorder (HADS-A ≥8).

*Abbreviations*: *EQ-5D-3L, European Quality of life 5-Dimensions 3-Levels; HADS, Hospital Anxiety and Depression Scale; HAQ-DI, Health Assessment Questionnaire–Disability Index; NRS, Numeric Rating Scale; and VAS, Visual Analogue Scale.*

**Supplementary Figure S6.** Health-related quality of life for complete cases within the age groups and the general Dutch population. **(A)** Mean health-related quality of life scores at baseline; **(B)** after 1 year; and **(C)** after 2 years, measured with the short form-36, stratified for age-group and compared to the general Dutch population norms.

*Abbreviation: HRQoL, Health-related quality of life.*

**Supplementary Table S3. Clinical and patient-reported outcomes over the 2-year follow-up period with EORA defined as age ≥70 years.**

|  | **<45 years**  **(n=119)** | **45-69 years**  **(n=245)** | **≥70 years**  **(n=61)** |
| --- | --- | --- | --- |
| **Clinical outcomes** |  | |  |
| - DAS44*, mean diff (95% CI)* | 0 (-0.2 to 0.2) | 0.1 (-0.1 to 0.3) | Ref |
| - bDMARD use*, OR (95% CI)* | 2.8 (0.7 to 12.2) | 4 (1.1 to 14.3)^1^ | Ref |
| - Remission*, OR (95% CI)* | 0.6 (0.4 to 1.1) | 0.5 (0.3 to 0.8)^2^ | Ref |
| - mTSS*, mean diff (95% CI)* | -2.7 (-3.8 to -1.7)^3^ | -2.3 (-3.2 to -1.4)^3^ | Ref |
| - JSN | -1.2 (-1.9 to -0.5)^3^ | -0.9 (-1.5 to -0.3)^2^ | Ref |
| - ES | -1.3 (-1.9 to -0.7)^3^ | -1.1 (-1.6 to -0.6)^3^ | Ref |
| **Patient-reported outcomes** |  | |  |
| - Pain (NRS)*, mean diff (95% CI)* | **1.2 (0.6 to 1.9)^3^** | 0.7 (0.1 to 1.2)^1^ | Ref |
| - Fatigue (VAS), *mean diff (95% CI)* | **17.1 (10.1 to 24.1)^3^** | 4.8 (-1.2 to 10.8) | Ref |
| - Functional ability (HAQ-DI)*, mean diff (95% CI)* | -0.02 (-0.19 to 0.14) | -0.08 (-0.22 to 0.06) | Ref |
| - Quality of life (EQ-5D-3L), *mean diff (95% CI)* | -0.03 (-0.08 to 0.02) | -0.02 (-0.06 to 0.02) | Ref |
| - Depression (HADS)*, OR (95% CI)* | 4.4 (1.2 to 15.4)^1^ | 2.3 (0.8 to 6.6) | Ref |
| - Anxiety (HADS)*, OR (95% CI)* | 5.2 (1.1 to 23.9)^1^ | 1.5 (0.4 to 5.4) | Ref |
| - PCS (SF-36)*, mean diff (95% CI)* | -0.7 (-3.4 to 2.1) | 0.1 (-2.3 to 2.4) | Ref |
| - MCS (SF-36)*, mean diff (95% CI)* | -2.9 (-5.7 to 0)^1^ | -1.7 (-4.1 to 0.7) | Ref |

^1^p < 0.05, ^2^p < 0.01, ^3^p < 0.001

The differences that exceed the MCID and are, thus, clinically relevant are shown in bold.

All analyses were adjusted for sex, number of comorbidities, symptom duration, time, ACPA positivity, and baseline DAS44 (except for DAS44) and mTSS (except for mTSS).

*Abbreviations: ACPA, anti-citrullinated protein antibody; bDMARD, biologic disease-modifying antirheumatic drug; DAS44, disease activity score 44 with 4 items (swollen joint count 44, tender joint count 53, erythrocyte sedimentation rate (ESR), general health (Visual Analogue Scale (VAS) 0-100 mm)); diff, difference; EORA, Elderly-onset rheumatoid arthritis; EQ-5D-3L, European Quality of life 5-Dimensions 3-Levels; ES, Erosion Score; HADS, Hospital Anxiety and Depression Scale; HAQ-DI, Health Assessment Questionnaire–Disability Index; JSN, Joint Space Narrowing; MCS, Mental Component Scale; MCID, minimal clinically important difference; mTSS, modified Total Sharp Score; OR, odds ratio; PCS, Physical Component Scale; Ref, reference group; SF-36, 36-item Short Form Health Survey; and VAS, Visual Analogue Scale.*

**Supplementary Table S4. Outcomes per timepoint with EORA defined as age ≥70 years.**

|  | **Time** | **<45 years**  (n= 119) | **45-69 years**  (n= 245) | **≥70 years** (n = 61) |
| --- | --- | --- | --- | --- |
| **Clinical outcomes** | | | | |
| **DAS44**, *mean (sd)* | T12 | 1.6 (0.9) | 1.7 (0.9) | 1.6 (0.8) |
|  | T24 | 1.6 (0.9) | 1.7 (0.8) | 1.5 (0.8) |
| **LDA,** *n (%)* | T12 | 78 (80) | 177 (82) | 44 (83) |
| (DAS44 ≤ 2.4) | T24 | 67 (84) | 144 (82) | 36 (88) |
| **Remission,** *n (%)* | T12 | 52 (53) | 109 (51) | 31 (58) |
| (DAS44 < 1.6) | T24 | 44 (55) | 94 (54) | 22 (54) |
| **bDMARD use,** *n (%)* | T12 | 38 (34) | 68 (29) | 11 (19) |
|  | T24 | 32 (30) | 61 (28) | 7 (14) |
| **mTSS,** *median (IQR)* | T12* | 0 (0 – 1) | 0.5 (0 – 2) | 1 (0 – 5) |
|  | T24 | 0 (0 – 0) | 0 (0 – 7) | 0 (0 – 1) |
| **PROs** | | | | |
| **Pain (NRS),** *median (IQR)*  *MCID ≥1* | T12 | 2 (1 – 6) | 2 (0 – 4) | 2 (0 – 3) |
|  | T24 | 2 (0 – 5) | 2 (0 – 5) | 2 (1 – 5) |
| **Fatigue (VAS),** *median (IQR)*  *MCID ≥10* | T12* | **54 (25 – 73)** | **36 (19 – 66)** | **31 (12 – 65)** |
|  | T24 | 44 (11 – 70) | 33 (18 – 64) | 34 (21 – 56) |
| **Functional ability (HAQ-DI),** *median (IQR)*  *MCID ≥0.22* | T12 | 0.5 (0 – 1.12) | 0.38 (0 – 1) | 0.6 (0.19 – 1.12) |
|  | T24 | 0.5 (0 – 1) | 0.62 (0 – 1) | 0.62 (0.12 – 1.38) |
| **Quality of life (EQ-5D-3L),** *mean (sd)*  *MCID ≥0.04* | T12 | 0.78 (0.15) | 0.77 (0.17) | 0.76 (0.16) |
|  | T24 | 0.81 (0.13) | 0.78 (0.18) | 0.76 (0.18) |
| **Depression (HADS-D≥8),** *n (%)* | T12 | 9 (11) | 30 (15) | 6 (13) |
|  | T24 | 5 (8) | 18 (11) | 6 (18) |
| **Anxiety (HADS-A≥8),** *n (%)* | T12 | 16 (19) | 34 (17) | 9 (20) |
|  | T24 | 9 (14) | 25 (16) | 4 (12) |
| **PCS (SF-36),** *mean (sd)*  *MCID ≥3-5* | T12 | 43 (11) | 43 (10) | 41 (11) |
|  | T24 | 44 (10) | 44 (10) | 41 (11) |
| **MCS (SF-36),** *mean (sd)*  *MCID ≥3-5* | T12 | 52 (9) | 53 (9) | 52 (11) |
|  | T24 | 53 (8) | 53 (10) | 54 (9) |

*p < 0.05 was considered significant.

The differences that exceed the MCID and are, thus, clinically relevant are shown in bold.

*Abbreviations: bDMARD, biologic disease-modifying antirheumatic drug; DAS44, disease activity score 44 with 4 items (swollen joint count 44, tender joint count 53, erythrocyte sedimentation rate, general health (VAS of 100 mm)); EORA, Elderly-onset rheumatoid arthritis; EQ-5D-3L, European Quality of life 5-Dimensions 3-Levels; HADS, Hospital Anxiety and Depression Scale; HAQ-DI, Health Assessment Questionnaire–Disability Index; IQR, interquartile range; MCID, minimal clinically important difference; MCS, Mental Component Scale; mTSS, modified Total Sharp Score; NRS, Numeric Rating Scale; PCS, Physical Component Scale; sd, standard deviation; SF-36, 36-item Short Form Health Survey; and VAS, Visual Analogue Scale.*

**Supplementary Figure S7.** Clinical outcomes during the 2-year follow-up period with EORA defined as age ≥70 years. **(A)** Mean DAS44 score; **(B)** Proportion of patients using a bDMARD; **(C)** Proportion of patients in DAS44 remission; **(D)** Estimated mean mTSS (114 patients under 45 years, 241 patients between 45 and 69 years, and 60 patients are 70 years or older), adjusted for sex, number of comorbidities, symptom duration, time, ACPA positivity, and baseline DAS44.

*Abbreviations: ACPA, anti-citrullinated protein antibody; DAS44, disease activity score 44 with 4 items (swollen joint count 44, tender joint count 53, erythrocyte sedimentation rate (ESR), general health (Visual Analogue Scale (VAS) 0-100 mm));* *EORA, Elderly-onset rheumatoid arthritis; and mTSS, modified Total Sharp Score.*

**Supplementary Figure S8.** Patient-reported outcomes during the 2-year follow-up period with EORA defined as age ≥70 years. **(A)** Mean pain (NRS); **(B)** Mean fatigue (VAS); **(C)** Mean functional ability (HAQ-DI); **(D)** Mean quality of life (EQ-5D-3L). **(E)** % of patients with a possible depression (HADS-D ≥8); and **(F)** % of patients with a possible anxiety disorder (HADS-A ≥8).

*Abbreviations: EORA, Elderly-onset rheumatoid arthritis; EQ-5D-3L, European Quality of life 5-Dimensions 3-Levels; HADS, Hospital Anxiety and Depression Scale; HAQ-DI, Health Assessment Questionnaire–Disability Index; NRS, Numeric Rating Scale; and VAS, Visual Analogue Scale.*

**Supplementary Figure S9.** Health-related quality of life within the age groups and the general Dutch population with EORA defined as age ≥70 years. **(A)** Mean health-related quality of life scores at baseline; **(B)** after 1 year; and **(C)** after 2 years, measured with the short form-36, stratified for age-group and compared to the general Dutch population norms.

*Abbreviation: EORA, Elderly-onset rheumatoid arthritis; and HRQoL, Health-related quality of life.*

**Supplementary Table S5. Clinical and patient-reported outcomes for autoantibody positive RA patients (either RF or ACPA positive) during the 2-year follow-up period stratified for age-group.**

|  | **<45 years**  **(n=79)** | **45-65 years**  **(n=127)** | **>65 years**  **(n=55)** |
| --- | --- | --- | --- |
| **Clinical outcomes** |  | |  |
| - DAS44*, mean diff (95% CI)* | -0.3 (-0.5 to -0.1)^1^ | -0.2 (-0.4 to 0) | Ref |
| - bDMARD use*, OR (95% CI)* | 1.1 (0.2 to 4.9) | 1.9 (0.5 to 6.6) | Ref |
| - Remission*, OR (95% CI)* | 1.4 (0.8 to 2.7) | 1 (0.6 to 1.8) | Ref |
| - mTSS*, mean diff (95% CI)* | -2.6 (-3.8 to -1.4)^3^ | -2.2 (-3.2 to -1.1)^3^ | Ref |
| - JSN | -1.4 (-2.3 to -0.5)^2^ | -1.3 (-2.1 to -0.5)^2^ | Ref |
| - ES | -1 (-1.6 to -0.4)^2^ | -0.7 (-1.2 to -0.2)^2^ | Ref |
| **Patient-reported outcomes** |  | |  |
| - Pain (NRS)*, mean diff (95% CI)* | 0.5 (-0.2 to 1.2) | 0.3 (-0.3 to 0.9) | Ref |
| - Fatigue (VAS), *mean diff (95% CI)* | **18.4 (10.8 to 25.9)^3^** | 8.1 (1.5 to 14.8)^1^ | Ref |
| - Functional ability (HAQ-DI)*, mean diff (95% CI)* | -0.09 (-0.26 to 0.08) | -0.11 (-0.26 to 0.04) | Ref |
| - Quality of life (EQ-5D-3L), *mean diff (95% CI)* | -0.02 (-0.07 to 0.03) | 0 (-0.05 to 0.04) | Ref |
| - Depression (HADS)*, OR (95% CI)* | 4.4 (0.9 to 21.2) | 3.3 (0.9 to 13) | Ref |
| - Anxiety (HADS)*, OR (95% CI)* | 7.6 (1.2 to 48.9)^1^ | 4 (0.8 to 20.1) | Ref |
| - PCS (SF-36)*, mean diff (95% CI)* | 0.7 (-2.2 to 3.5) | 0.7 (-1.8 to 3.2) | Ref |
| - MCS (SF-36)*, mean diff (95% CI)* | -2.5 (-5.4 to 0.5) | -1.4 (-4 to 1.1) | Ref |

^1^p < 0.05, ^2^p < 0.01, ^3^p < 0.001

The differences that exceed the MCID and are, thus, clinically relevant are shown in bold.

All analyses were adjusted for sex, number of comorbidities, symptom duration, time, and baseline DAS44 (except for DAS44) and mTSS (except for mTSS).

*Abbreviations: ACPA, anti-citrullinated protein antibody; bDMARD, biologic disease-modifying antirheumatic drug; DAS44, disease activity score 44 with 4 items (swollen joint count 44, tender joint count 53, erythrocyte sedimentation rate (ESR), general health (Visual Analogue Scale (VAS) 0-100 mm)); diff, difference; EQ-5D-3L, European Quality of life 5-Dimensions 3-Levels; ES, Erosion Score; HADS, Hospital Anxiety and Depression Scale; HAQ-DI, Health Assessment Questionnaire–Disability Index; JSN, Joint Space Narrowing; MCS, Mental Component Scale; MCID, minimal clinically important difference; mTSS, modified Total Sharp Score; OR, odds ratio; PCS, Physical Component Scale; Ref, reference group; RF, Rheumatoid Factor; SF-36, 36-item Short Form Health Survey; and VAS, Visual Analogue Scale.*

**Supplementary Table S6. Outcomes per timepoint for autoantibody positive RA patients (either RF or ACPA positive) stratified for age-group.**

|  | **Time** | **<45 years**  (n= 79) | **45-65 years**  (n= 127) | **>65 years** (n = 55) |
| --- | --- | --- | --- | --- |
| **Clinical outcomes** | | | | |
| **DAS44**, *mean (sd)* | T12 | 1.4 (0.9) | 1.5 (0.8) | 1.7 (0.8) |
|  | T24 | 1.5 (0.9) | 1.6 (0.9) | 1.7 (0.8) |
| **LDA,** *n (%)* | T12 | 58 (84) | 101 (88) | 36 (77) |
| (DAS44 ≤ 2.4) | T24 | 45 (83) | 81 (86) | 31 (84) |
| **Remission,** *n (%)* | T12 | 40 (58) | 63 (55) | 26 (55) |
| (DAS44 < 1.6) | T24 | 32 (59) | 50 (53) | 21 (57) |
| **bDMARD use,** *n (%)* | T12 | 23 (30) | 37 (31) | 14 (26) |
|  | T24 | 20 (29) | 33 (30) | 8 (17) |
| **mTSS,** *median (IQR)* | T12* | 0.5 (0 – 1.5) | 1 (0 – 2.5) | 3 (1 – 8) |
|  | T24 | 0 (0 – 0.3) | 0 (0 – 1) | 0 (0 – 1) |
|  | | | | |
| **Pain (NRS),** *median (IQR)*  *MCID ≥1* | T12 | 1 (0 – 5) | 2 (0 – 3) | 1 (0 – 3) |
|  | T24 | 1 (0 – 5) | 1 (0 – 5) | 2 (1 – 5) |
| **Fatigue (VAS),** *median (IQR)*  *MCID ≥10* | T12 | 41 (23 – 70) | 36 (19 – 66) | 30 (11 – 51) |
|  | T24 | 32 (10 – 67) | 38 (20 – 66) | 29 (17 – 52) |
| **Functional ability (HAQ-DI),** *median (IQR)*  *MCID ≥0.22* | T12 | 0.25 (0 – 0.75) | 0.25 (0 – 0.75) | 0.25 (0 – 1.12) |
|  | T24 | 0.5 (0 – 0.75) | 0.38 (0 – 0.88) | 0.38 (0 – 1) |
| **Quality of life (EQ-5D-3L),** *mean (sd)*  *MCID ≥0.04* | T12 | 0.8 (0.17) | 0.79 (0.14) | 0.77 (0.16) |
|  | T24 | 0.82 (0.14) | 0.78 (0.19) | 0.79 (0.12) |
| **Depression (HADS-D≥8),** *n (%)* | T12 | 6 (11) | 12 (11) | 2 (5) |
|  | T24 | 2 (5) | 9 (11) | 4 (13) |
| **Anxiety (HADS-A≥8),** *n (%)* | T12 | 12 (21) | 18 (17) | 3 (7) |
|  | T24 | 5 (11) | 14 (18) | 3 (10) |
| **PCS (SF-36),** *mean (sd)*  *MCID ≥3-5* | T12 | 45 (11) | 44 (10) | 42 (11) |
|  | T24 | 46 (9) | 45 (11) | 45 (10) |
| **MCS (SF-36),** *mean (sd)*  *MCID ≥3-5* | T12 | 52 (9) | 53 (9) | 52 (10) |
|  | T24 | 53 (7) | 52 (10) | 55 (7) |

*p < 0.05 was considered significant.

The differences that exceed the MCID and are, thus, clinically relevant are shown in bold.

*Abbreviations: ACPA, anti-citrullinated protein antibody; bDMARD, biologic disease-modifying antirheumatic drug; DAS44, disease activity score 44 with 4 items (swollen joint count 44, tender joint count 53, erythrocyte sedimentation rate, general health (VAS of 100 mm)); EQ-5D-3L, European Quality of life 5-Dimensions 3-Levels; HADS, Hospital Anxiety and Depression Scale; HAQ-DI, Health Assessment Questionnaire–Disability Index; IQR, interquartile range; MCID, minimal clinically important difference; MCS, Mental Component Scale; mTSS, modified Total Sharp Score; NRS, Numeric Rating Scale; PCS, Physical Component Scale; RF, Rheumatoid Factor; sd, standard deviation; SF-36, 36-item Short Form Health Survey; and VAS, Visual Analogue Scale.*

**Supplementary Figure S10.** Clinical outcomes for autoantibody positive RA patients (either RF or ACPA positive) during the 2-year follow-up period stratified for age-group. **(A)** Mean DAS44 score; **(B)** Proportion of patients using a bDMARD; **(C)** Proportion of patients in DAS44 remission; **(D)** Estimated mean mTSS (78 patients under 45 years, 126 patients between 45 and 65 years, and 55 patients older than 65 years), adjusted for sex, number of comorbidities, symptom duration, time, and baseline DAS44.

*Abbreviations: ACPA, anti-citrullinated protein antibody; DAS44, disease activity score 44 with 4 items (swollen joint count 44, tender joint count 53, erythrocyte sedimentation rate (ESR), general health (Visual Analogue Scale (VAS) 0-100 mm)); RF, Rheumatoid Factor; and mTSS, modified Total Sharp Score.*

**Supplementary Figure S11.** Patient-reported outcomes for autoantibody positive RA patients (either RF or ACPA positive) during the 2-year follow-up period stratified for age-group. **(A)** Mean pain (NRS); **(B)** Mean fatigue (VAS); **(C)** Mean functional ability (HAQ-DI); **(D)** Mean quality of life (EQ-5D-3L). **(E)** % of patients with a possible depression (HADS-D ≥8); and **(F)** % of patients with a possible anxiety disorder (HADS-A ≥8).

*Abbreviations*: *ACPA, anti-citrullinated protein antibody; EQ-5D-3L, European Quality of life 5-Dimensions 3-Levels; HADS, Hospital Anxiety and Depression Scale; HAQ-DI, Health Assessment Questionnaire–Disability Index; NRS, Numeric Rating Scale; RF, Rheumatoid Factor; and VAS, Visual Analogue Scale.*

**Supplementary Figure S12.** Health-related quality of life for autoantibody positive RA patients (either RF or ACPA positive) within the age groups and the general Dutch population. **(A)** Mean health-related quality of life scores at baseline; **(B)** after 1 year; and **(C)** after 2 years, measured with the short form-36, stratified for age-group and compared to the general Dutch population norms.

*Abbreviation: ACPA, anti-citrullinated protein antibody; HRQoL, Health-related quality of life; and RF, Rheumatoid Factor.*
